# Control of nitrogen fixation and ammonia excretion in *Azorhizobium caulinodans*

**DOI:** 10.1371/journal.pgen.1010276

**Published:** 2022-06-21

**Authors:** Timothy Lyndon Haskett, Ramakrishnan Karunakaran, Marcelo Bueno Batista, Ray Dixon, Philip Simon Poole

**Affiliations:** 1 Department of Plant Sciences, University of Oxford, Oxford, United Kingdom; 2 Department of Molecular Microbiology, John Innes Centre, Norwich, United Kingdom; Universidad de Sevilla, SPAIN

## Abstract

Due to the costly energy demands of nitrogen (N) fixation, diazotrophic bacteria have evolved complex regulatory networks that permit expression of the catalyst nitrogenase only under conditions of N starvation, whereas the same condition stimulates upregulation of high-affinity ammonia (NH_3_) assimilation by glutamine synthetase (GS), preventing excess release of excess NH_3_ for plants. Diazotrophic bacteria can be engineered to excrete NH_3_ by interference with GS, however control is required to minimise growth penalties and prevent unintended provision of NH_3_ to non-target plants. Here, we tested two strategies to control GS regulation and NH_3_ excretion in our model cereal symbiont *Azorhizobium caulinodans Ac*LP, a derivative of ORS571. We first attempted to recapitulate previous work where mutation of both P_II_ homologues *glnB* and *glnK* stimulated GS shutdown but found that one of these genes was essential for growth. Secondly, we expressed unidirectional adenylyl transferases (uATs) in a Δ*glnE* mutant of *Ac*LP which permitted strong GS shutdown and excretion of NH_3_ derived from N_2_ fixation and completely alleviated negative feedback regulation on nitrogenase expression. We placed a *uAT* allele under control of the NifA-dependent promoter P*nifH*, permitting GS shutdown and NH_3_ excretion specifically under microaerobic conditions, the same cue that initiates N_2_ fixation, then deleted *nifA* and transferred a rhizopine *nifA*_*L94Q/D95Q*_*-rpoN* controller plasmid into this strain, permitting coupled rhizopine-dependent activation of N_2_ fixation and NH_3_ excretion. This highly sophisticated and multi-layered control circuitry brings us a step closer to the development of a "synthetic symbioses” where N_2_ fixation and NH_3_ excretion could be specifically activated in diazotrophic bacteria colonising transgenic rhizopine producing cereals, targeting delivery of fixed N to the crop while preventing interaction with non-target plants.

## Author summary

Inoculation of cereal crops with associative diazotrophic bacteria that convert atmospheric nitrogen (N_2_) into ammonia (NH_3_) could be used to sustainably improve delivery of nitrogen to crops. However, due to the costly energy demands of N_2_ fixation, bacteria restrict excess production of NH_3_ and release to the plants. Diazotrophs can be engineered for excess NH_3_ production and release, however genetic control is required to minimise growth penalties and prevent unintended provision of NH_3_ to non-target weed species. Here, we engineer coupled control of N_2_ fixation and NH_3_ release in response to the signalling molecule rhizopine supplemented *in vitro*. This control circuitry represents a prototype for the future development of a “synthetic symbiosis” where bacterial N_2_ fixation and NH_3_ excretion could be specifically activated following colonisation of transgenic rhizopine producing cereals in the field, minimising bacterial energy requirements and preventing provision of NH_3_ to non-target plants.

## Introduction

Nitrogen (N) is an essential constituent of all biological organisms, but metabolically accessible forms are scarce in most environments [1], restricting biomass production. In agriculture, productivity of cereal crops, which are a staple of human dietary requirements, requires large-scale supplementation with synthetic N fertilisers to meet global food security requirements [2]. However, synthesis and excessive application of N fertilisers has a large energy cost, causes CO_2_ release and results in loss of reduced N to the environment, which has doubled reactive N in the atmosphere and polluted waterways causing eutrophication and oxygen-depleted dead zones [3]. In contrast, N fertilisers are largely unaffordable to small-hold farmers in developing countries such as those in Sub-Saharan Africa [4], restricting yields to a fraction of their maximum potential [5]. Inoculation of cereals with root-associative diazotrophic bacteria that convert atmospheric N_2_ gas to ammonia (NH_3_) through the action of oxygen-labile nitrogenase represents an affordable and sustainable alternative to the use of N fertilisers in agriculture [6–8]. Although associative diazotrophs have been estimated to fix up to 25 kg N ha^-1^ year^-1^ in agricultural systems [9], responses to inoculation are typically inconsistent due to sub-optimal competitiveness for root colonisation and persistence in soil [10–13]. Furthermore, due to the costly energy demands of N_2_ fixation, which consumes at least 16 mol ATP per mol N_2_ fixed *in vitro*, bacteria have evolved complex regulatory networks that permit expression and activity of the N_2_-fixing catalyst nitrogenase only under conditions of N starvation, whereas the same condition stimulates upregulation of high-affinity NH_3_ assimilation by glutamine synthetase (*glnA*, GS), preventing excess release of excess NH_3_ for plants [14,15].

Associative diazotrophic bacteria can been engineered for excess production and excretion of NH_3_ by several strategies [14,16,17]. For example, in *Azotobacter vinelandii*, insertional inactivation of *nifL*, which encodes an oxygen as well as N and carbon sensing anti-activator of the nitrogenase master regulator NifA, drives constitutive nitrogenase activity resulting in excretion of NH_3_ from the cell [18–21]. The same effect was achieved by expressing mutant *nifA* alleles that are resistant to inhibition by NifL [19,22,23]. While excess NH_3_ production itself is likely to activate regulatory feedback mechanisms reducing GS biosynthetic activity and NH_3_ assimilation [16], mutating *glnA* [24–28] or genes involved in GS regulation may also be required to inhibit NH_3_ assimilation more strongly and favour optimal NH_3_ excretion [29,30].

Bacterial GS belongs to the “class I” type enzymes comprised of 12 identical subunits which are each adenylylated or deadenylated by a bidirectional adenylyl transferase (AT, encoded by *glnE*) at the Tyr_397_ residue, with the fully deadenylylated GS form being biosynthetically active and vice versa [31]. Directionality of the ATase reaction is regulated by the post-translational modification state of P_II_ signal transduction proteins [32]. The activity of P_II_ proteins is regulated by uridylylation/deuridylylation by the bidirectional uridylyltransferase (UT) GlnD which represents the most basal regulator in the cascade and can directly sense N status of the cell [33]. GlnD uridylylates P_II_ under conditions of N-starvation and the resulting P_II_-UMP ultimately triggers dephosphorylation of ATase and hence deadenylylation and activation of GS [34]. In *Azorhizobium caulinodans* (*Ac*), insertional inactivation of both P_II_ homologues *glnB* and *glnK* produced a mutant that was unable to activate GS by deadenylylation, driving NH_3_-insensitive N_2_ fixation and excretion of NH_3_ into the growth media [29]. Critically, this engineering strategy does not appear to be universally applicable as P_II_ is essential for NifA and nitrogenase activity in some bacteria [35,36], whereas it is essential for growth in others [37,38]. In a *ΔglnE* ATase mutant of *Azospirillum brasilense*, complementation with unidirectional adeyltransferase (uAT) alleles that encoded only the C-terminal adenylylation domain [32] drove strong adenylylation of GS resulting in excretion of NH_3_ into the growth media [30]. This strategy likely represents a more universally applicable approach for engineering NH_3_ excretion in diazotrophs because the ATase is highly conserved, has a specific function, and can be readily mutated across diverse diazotrophic bacterial taxa [16,39–41], albeit the mutation appears to be lethal in the heterotroph *Mycobacterium tuberculosis* [42,43].

From an agricultural perspective, there are three major caveats of engineering diazotrophic bacteria for excessive production and excretion of NH_3_; i) uncontrolled *nifA* and (or) nitrogenase expression has a severe energy burden on the cell that could abolish competitiveness for root colonisation; ii) interference with GS activity typically renders strains auxotrophic for the essential amino acid glutamine, which could further reduce competitiveness; and iii) NH_3_ excreting bacteria have potential to supply NH_3_ to non-target weed species following promiscuous colonisation in the field. Therefore, establishing control of N_2_ fixation and NH_3_ excretion will be crucial for the optimisation of strains as agricultural inoculants. Control of NH_3_ excretion has already been achieved in *A*. *vinelandii* by establishing IPTG-dependent expression of *glnA* [28], and in *A*. *brasilense* by establishing anhydro-tetracycline inducible expression of uATs [30,44]. However, use of plant-derived signals to control N_2_-fixation and NH_3_ excretion would be far more applicable in the environment and could impart partner-specificity to target delivery of fixed N to crops and prevent interactions with non-target host plants following promiscuous colonisation [45,46].

**Fig 1:**
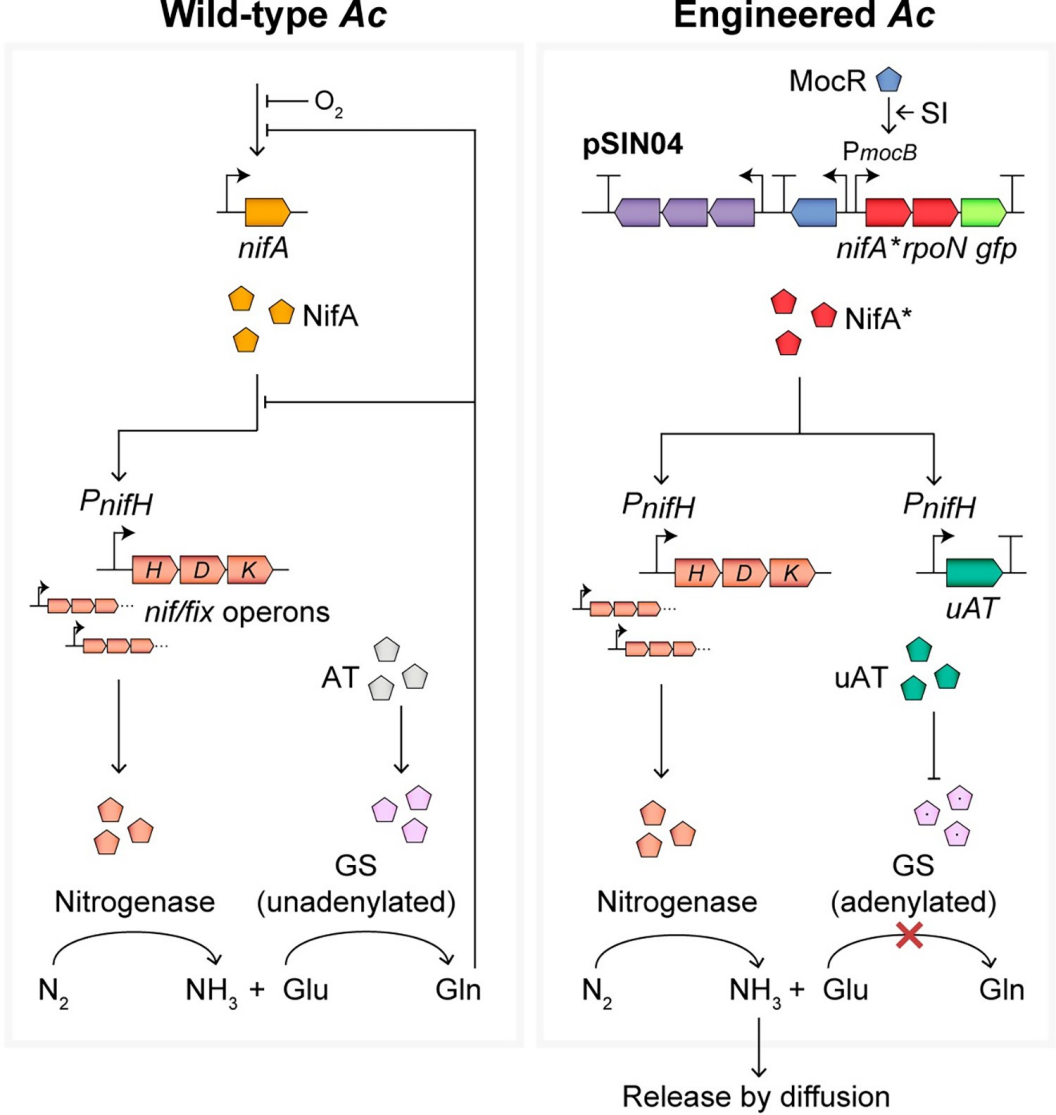
Model for rhizopine control of nitrogen fixation and ammonia excretion in engineered *Ac*LP. In the wild-type bacterium, NifA is activated under N_2_-fixing (N-free microaerobic) conditions leading to transcription of nitrogenase (*nif* and *fix*) genes and subsequently N_2_ fixation. Under the same conditions, the bidirectional adenylyl transferase (AT, encoded by *glnE*) activates glutamine synthetase (GS) by deadenylylation. GS catalyses assimilation of NH_3_ via the conversion of glutamate (Glu) to glutamine (Gln), which feeds back to repress nitrogenase expression and NifA activity, preventing excess production and release of NH_3_ from the cell. Our engineered strain is a *ΔglnE ΔnifA* mutant carries a rhizopine-inducible *nifA*_*L94Q/D95Q*_*-rpoN* cassette that drives nitrogenase expression and N_2_ fixation in response to addition of the rhizopine *scyllo-*inosamine (SI). Rhizopine-inducible expression is activated from the promoter P*mocB* by the transcription factor MocR bound to SI. Additionally, the strain carries a mini-Tn*7* integrated nitrogenase promoter (P*nifH*) driving expression of a unidirectional adenylyltransferase (uAT) under NifA control. Thus, uAT expression is activated in the presence of SI and drives shutdown of GS by adenylylation, preventing assimilation of NH_3_ derived from N_2_ fixation. Because shutdown of GS prevents glutamine biosynthesis, repression on nitrogenase expression and NifA activity is also alleviated. The combined effects of NH_3_-insensitive nitrogenase expression and abolished NH_3_ assimilation results in release of NH_3_ from the cell by diffusion.

We previously developed synthetic rhizopine signalling between barley and the model endophyte *Azorhizobium caulinodans Ac*LP that stimulates transcriptional activation of the mutant nitrogenase master regulator *nifA*_*L94Q/D95Q*,_ which partially escapes nitrogen regulation, and when paired with the sigma factor RpoN drives N_2_ fixation in bacteria colonising rhizopine producing (*RhiP*) barley roots [45,47,48]. Here, we demonstrate that wild-type and engineered *Ac* strains do not release fixed N as NH_3_ into the growth media when cultured under N_2_-fixing conditions and therefore sought to engineer this trait by interfering with high-affinity NH_3_ assimilation catalysed by GS. In our attempts to recapitulate NH_3_ excreting *glnB glnK* double mutants of *Ac*LP [29], we found that deletion of both P_II_ homologues was only possible when second copy of *glnB* was first integrated into the chromosome suggesting one of the P_II_ homologues were essential for growth. GS and nitrogenase activity in the resulting strain exhibited minimal variation from that of the wild-type, but nevertheless the strain excreted low levels of NH_3_ into the growth media. To optimise rates of NH_3_ excretion, we utilised a second engineering strategy where a *Ac*LP*ΔglnE* mutant was complemented with uATs. In congruency with similar experiments performed in *A*. *brasilense* [30], uAT expression drove strong shutdown of GS, but also completely alleviated negative feedback inhibition of nitrogenase by NH_3_ and stimulated NH_3_ excretion. By placing uAT expression under control of NifA, we established control of these traits in response to microaerobic conditions, the same cue that initiates N_2_ fixation, then transferred rhizopine control of *nifA*_*L94Q/D95Q-*_*rpoN* into this strain linking activation of N_2_-fixation and NH_3_ excretion (Fig 1). This highly sophisticated control circuitry represents a significant milestone in the development of a “synthetic symbiosis” where N_2_ fixation and NH_3_ excretion could be activated in bacteria specifically colonising target rhizopine producing cereals, targeting delivery of N to the crops while avoiding potential interactions with non-target plants.

## Results

### Deletion or strong repression of the *P*_*II*_ genes is lethal

**Fig 2:**
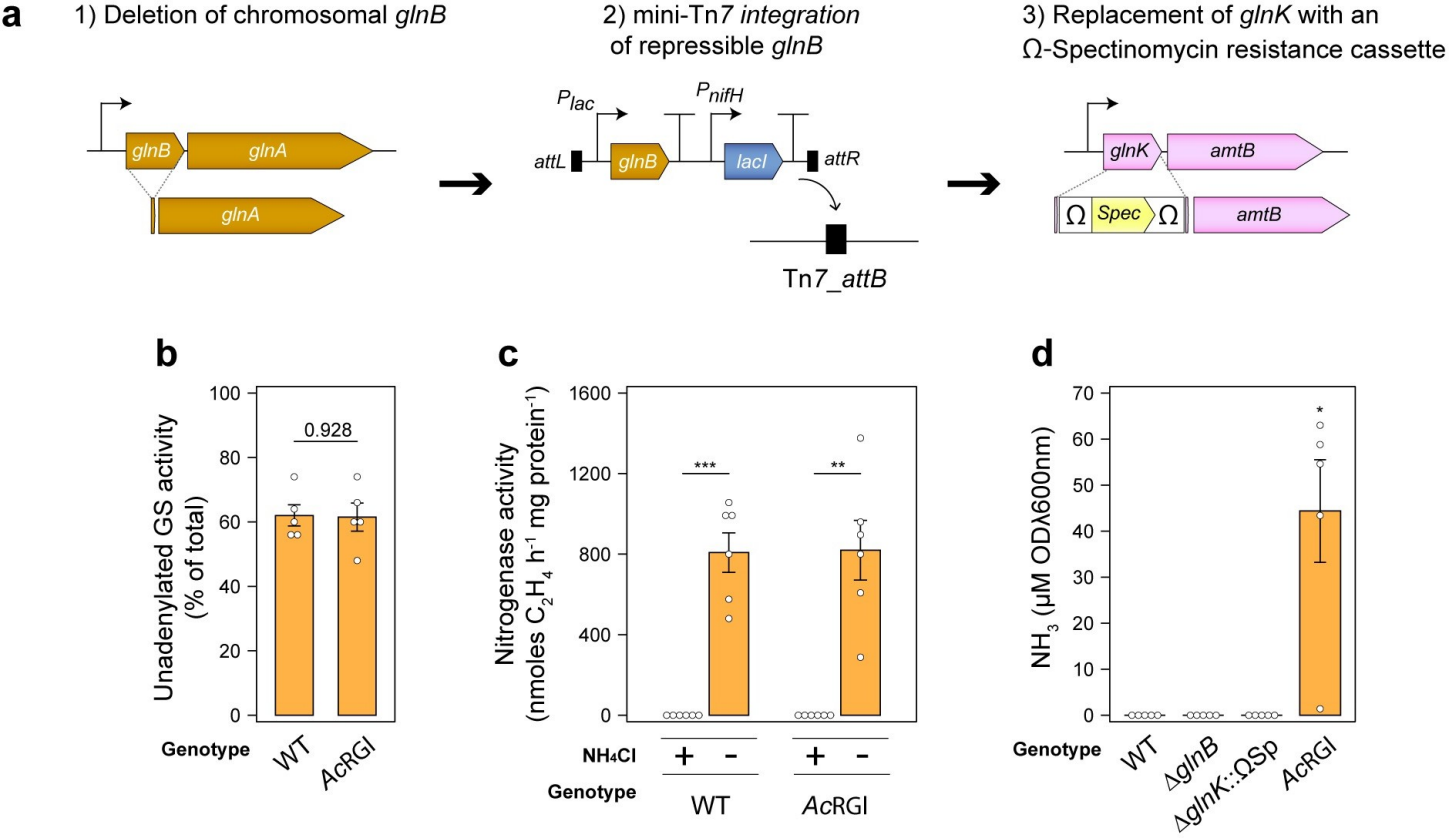
Strong repression of *glnB* in a *glnK* mutant has minimal effect on glutamine synthetase and nitrogenase activity but drives low-level ammonia excretion. **(a)** Strategy for generating strain *Ac*RGl with the double *ΔglnB* and *ΔglnK*::ΩSp mutation following integration of an IPTG-derepressible *glnB* gene into the chromosome of *Ac*LP. **(b)** Activity of the unadenylylated (active) form of GS in *n = 5* wild-type (WT) or *Ac*RGl cultures incubated for 24-h as determined by γ-glutamyl transferase assays in the presence or absence of 60 mM MgCl_2_ (see S2 Fig for total activity). **(c)** Nitrogenase activity measured by acetylene reduction in *n = 6* cultures between 3-h– 21-h **(d)** Spectrophotometric determination of NH_3_ in media of *n = 5* cultures grown for 24-h. Cultures for all assays were grown in N_2_-fixing conditions (N-free UMS media with 3% O_2_ in the headspace). Error bars represent one SEM. Independent two-tailed students t-tests were used to compare means. Exact P-values are provided where P > 0.05. *P < 0.05, **P < 0.01, ***P < 0.001. The wild-type *Ac*LP was used as a reference group for comparison of means in panel (d).

It was previously demonstrated that insertional inactivation of the *Ac P*_*II*_ genes *glnB* and *glnK* stimulates shutdown of GS by adenylylation and alleviates negative feedback inhibition of nitrogenase by the product NH_3_, preventing NH_3_ assimilation and favouring excretion into the growth media [29]. We attempted to recapitulate these experiments in *Ac*LP, a derivative of *Ac* harbouring a mini-Tn7 *attB* integration site stably recombined into its chromosome, by constructing a markerless deletion of *glnB* and replacing *glnK* with an omega (Ω)-spectinomycin resistance (Sp) cassette. Although the single *ΔglnB* and *ΔglnK*::ΩSp mutations were readily acquired, we were unable to acquire the double mutant by introduction of the *ΔglnK*::ΩSp mutation into *Ac*LP*ΔglnB* when selection was performed on rich or minimal media supplemented with glutamine as a sole N source, suggesting the resulting phenotype was lethal. To explore this notion further, we integrated into the chromosome of *Ac*LP*ΔglnB* a construct encoding *glnB* with the strong ribosome binding site (RBS) RStd expressed from the IPTG derepressible promoter P*lac* (Fig 2A) and were subsequently able to acquire the *ΔglnB ΔglnK*::ΩSp double mutation when selection was performed on rich media in the absence of IPTG, confirming that one of the P_II_ proteins was essential for growth.

We next sought to test whether reduced translation of the introduced *glnB* gene would stimulate GS shutdown and NH_3_ excretion by tuning the ribosome binding site (RBS). Seven synthetic RBS’ were experimentally demonstrated to produce translation rates spanning two to three orders of magnitude (S1 Fig), but only when *glnB* was fused to the strongest RBS RStd and integrated into the *Ac*LP*ΔglnB* chromosome were we able to subsequently isolate the *ΔglnK*::ΩSp mutation (hereby termed strain *Ac*RGl), suggesting that *glnB* had been repressed as much as was tolerable. We assessed total GS specific activity and that of the unadenylylated active enzyme in *Ac*RGl by performing γ-glutamyl transferase assays on whole cells in the presence or absence of 60 mM MgCl_2_ which specifically inhibits the adenylylated enzyme [49], and found that mutant exhibited higher total GS activity compared to the wild-type (S2A Fig), presumably due to elevated *glnA* expression (S2B Fig) as is typical of *glnB* mutants [26,29], whereas the adenylylation state of GS (depicted here as percentage of unadenylylated GS activity) was unchanged (Fig 2B). We also found that the specific nitrogenase activity of strain *Ac*RGl was no different from that of the wild-type, being repressed by supplementation of 10 mM NH_4_Cl into the growth media (Fig 2C). Spectrophotometric quantification of NH_3_ was next performed using the indophenol method [50] on the strains grown for 24-h under N_2_-fixing conditions (here defined as N-free UMS with O_2_ in the headspace adjusted to 3%). No NH_3_ was detected in the wild-type or *ΔglnB* and *ΔglnK*::ΩSp single mutants, whereas we detected trace amounts of NH_3_ in the growth media of strain *Ac*RGl (Fig 2D). Given that construction of the *Ac*LP*ΔglnB ΔglnK*::ΩSp double mutant was lethal, and strong repression of *glnB* had minimal effect on GS and nitrogenase regulation permitting only low-level NH_3_ excretion, we concluded that these strategies were inadequate to establish control of NH_3_ excretion in *Ac*LP and opted to pursue an alternative strategy.

### uAT expression drives GS inactivation and ammonia excretion

**Fig 3:**
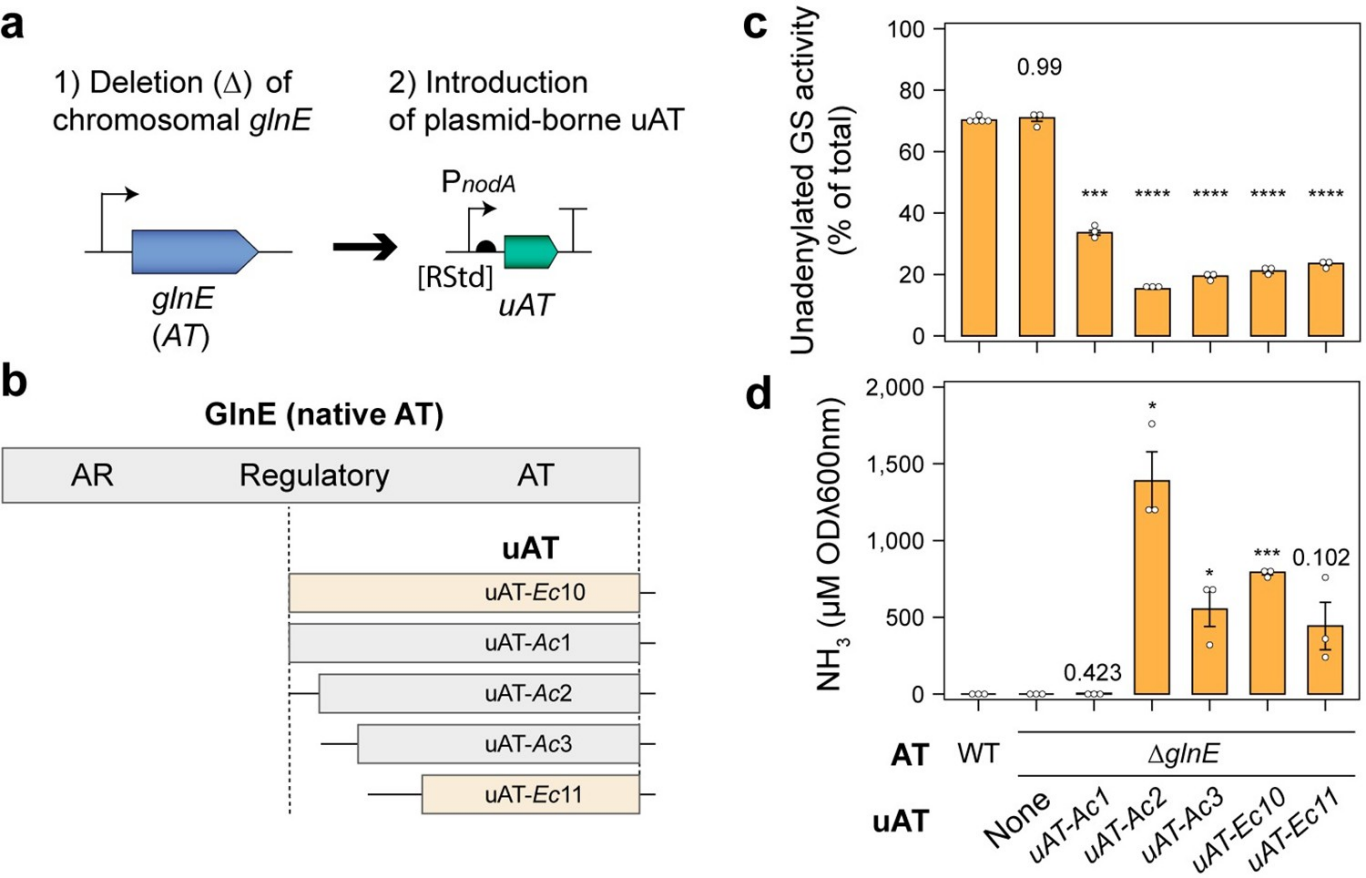
uAT expression drives GS adenylylation and ammonia excretion in a *ΔglnE* background. **(a)** Strategy for complementation of the *ΔglnE* mutation with naringenin-inducible unidirectional adenylyl transferases (uAT) expressed from low-copy parent plasmid pOPS1536. **(b)** A series of truncated uAT proteins harbouring the adenylyl transferase (AT) but not the adenylyl removase (AR) domain were used in this study. The *uAT-Ec10* and *uAT-Ec11* alleles are derived from *E*. *coli* and were described previously [30], whereas *uAT-Ac* alleles are derived from *Ac*LP. The nucleotide sequences for these alleles are provided in S1 File. **(c)** Activity of the unadenylylated (active) form of GS in *n = 5* for *Ac*LP (wild-type, WT) or *n* = 3 cultures incubated for 3-h in N_2_-fixing conditions (N-free UMS media with 3% O_2_ in the headspace) without the inducer naringenin as determined by γ-glutamyl transferase assays in the presence or absence of 60 mM MgCl_2_ (see S4 Fig for total activity). **(d)** Spectrophotometric determination of NH_3_ in media of *n =* 3 cultures grown for 24-h in N_2_-fixing conditions. Error bars represent one SEM. Independent two-tailed students t-tests with the Bonferroni-holm adjustment were used to compare means using the wild-type *Ac*LP as a reference group. Exact P-values are provided where P > 0.05. *P < 0.05, ***P < 0.001, ****P < 0.0001.

In a Δ*glnE* mutant of *A*. *brasilense*, controlled expression of a N-terminal truncated uAT consisting of only the AT adenylylation domain resulted in unidirectional activity driving strong inactivation of GS by adenylylation and excretion of NH_3_ into the growth media [30]. We recapitulated these experiments in a Δ*glnE* mutant of *Ac*LP by using the *Sinorhizobium meliloti* derived P*nodA* promoter (S3 Fig), to drive expression of a series of truncated uATs derived from *Ac* or those previously described for *E*. *coli* (Fig 3A and 3B) [30]. We assessed GS specific activity and that of the unadenylylated enzyme using γ-glutamyl transferase assays on cells grown in N_2_-fixing conditions for 3-h and confirmed that leaky non-induced uAT expression stimulated GS adenylylation (Fig 3C), while having minimal effect on total GS specific activity relative to wild-type bacteria (S4 Fig). The strains also excreted between 0.1–1.5 mM of NH_3_ after 24-h incubation in N_2_-fixing conditions, whereas the wild-type and Δ*glnE* mutant did not excrete detectable levels of NH_3_ (Fig 3D). Interestingly, we found that NH_3_ excretion was sub-optimal when the P*nodA* promoter controlling uAT expression was induced with 5 uM naringenin (S5 Fig), suggesting that strong *uAT* overexpression is metabolically detrimental, as was observed in *A*. *brasilense* [44]. This indicated that more finely tuned *uAT* expression would be critical to achieve stringent control of GS adenylylation in *Ac*LP.

### Shutdown of glutamine biosynthesis alleviates negative feedback on nitrogenase

**Fig 4:**
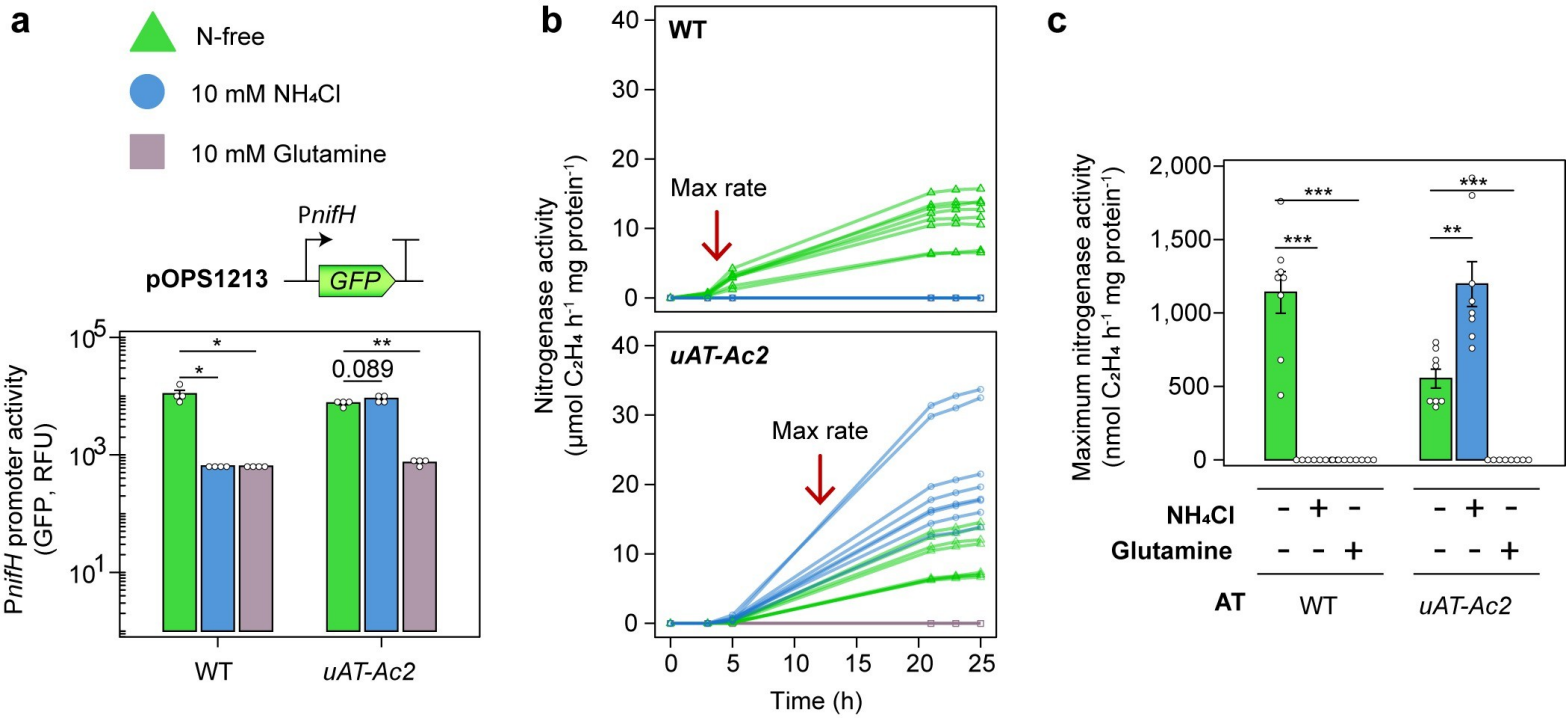
uAT expression abolishes negative feedback regulation on nitrogenase. **(a)** A P*nifH*::*GFP* reporter carried on plasmid pOPS1213 was mobilised into the wild-type (WT) *Ac*LP and *Ac*LP*ΔglnE* expressing *uAT-Ac2* on a second low-copy plasmid and induction was measured in *n =* 4 cultures grown for 24-h under the conditions indicated. Relative fluorescence units (RFU) are defined here as GFP fluorescence/OD600λnm **(b)** Nitrogenase activity was measured by acetylene reduction in *n =* 8 cultures grown under N_2_-fixing conditions (N-free UMS media with 3% O_2_ in the headspace) and **(c)** the maximum rates are presented. Error bars represent one SEM. Independent two-tailed students t-tests with Bonferroni-holm adjustment were used to compare means. Exact P-values are provided where P > 0.05. **P < 0.01, ***P < 0.001.

Expression of *uAT* restricts glutamine production via the high affinity GS-dependent NH_3_ assimilation pathway, providing us with a unique opportunity to tease apart the effects of NH_3_ and glutamine on the nitrogenase (*nif*) gene expression. We postulated that NH_3_ must first be converted into glutamine to mediate repression of *nif* genes and tested this hypothesis first by examining expression of P*nifH* fused to *GFP* on plasmid pOPS1213 in wild-type bacteria and in *Ac*LPΔ*glnE* expressing the *uAT*-*Ac2* allele from the non-induced P*nodA* promoter on a second plasmid. As expected, P*nifH*::*GFP* activity in both strains grown under microaerobic conditions (3% O_2_ in the headspace) was strongly repressed by supplementation with 10 mM glutamine however, while P*nifH*::*GFP* was repressed in the wild-type by supplementation with 10 mM NH_4_Cl, P*nifH*::*GFP* expression was not repressed by NH_4_Cl in *Ac*LPΔ*glnE* expressing *uAT-Ac2* (Fig 4A). We observed a similar pattern when nitrogenase activity was assessed by ARAs (Fig 4B and 4C), indicating that NH_3_ itself has no effect on negative feedback regulation of *nif* genes but must be converted into glutamine or potentially other amino acids to facilitate repression. Engineering NH_3_ excreting bacteria by targeted GS shutdown therefore has two advantages; i) alleviating negative feedback regulation of *nif* genes and ii) preventing NH_3_ assimilation to favour release.

### NifA control of *uAT* expression

**Fig 5:**
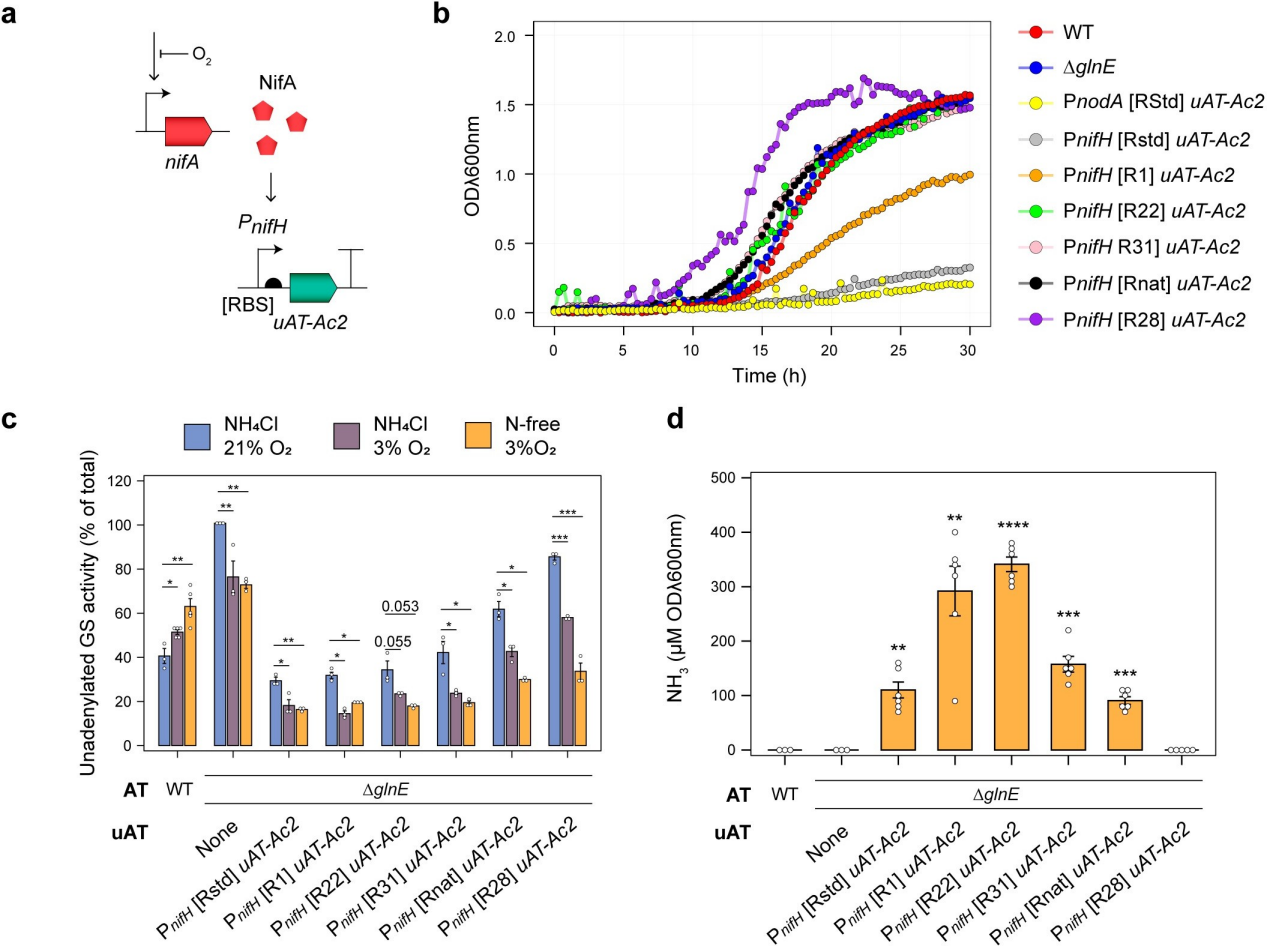
Coupled activation of nitrogen fixation and GS adenylylation via NifA-dependent expression of *uAT*. **(a)** Strategy for complementation of the *ΔglnE* mutation with NifA-inducible unidirectional adenylyl transferases (uAT) integrated into the chromosome using mini-Tn*7*. **(b)** Growth of control strains and those expressing uATs in UMS media supplemented with 20 mM succinate and 10 mM NH_4_Cl under aerobic conditions. See S6 Fig for full growth statistics. **(c)** Activity of the unadenylylated (active) form of GS in *n =* 5 for wild-type (WT) *Ac*LP or *n* = 3 cultures incubated for 24-h in as determined by γ-glutamyl transferase assays in the presence or absence of 60 mM MgCl_2_. (**d**) Spectrophotometric determination of NH_3_ in media of *n =* 3 WT and *ΔglnE* or *n* = 5 cultures grown for 24-h in N_2_-fixing conditions (N-free UMS media with 3% O_2_ in the headspace). Error bars represent one SEM. Independent two-tailed students t-tests with the Bonferroni-holm adjustment were used to compare means. Exact P-values are provided where P > 0.05. **P < 0.01, ***P < 0.001. The wild-type bacteria was used as a reference group for statistical comparisons in panel (d).

As a direct consequence of engineering NH_3_ excretion through GS interference, bacteria typically become auxotrophic for glutamine. While this may not be non-problematic for cultures grown *in vitro* under gnotobiotic conditions, glutamine auxotrophs in the field would be unable to compete or persist in the soil and rhizosphere. In rhizobia-legume symbioses, rhizobia only restrict NH_3_ assimilation after infecting the low-oxygen environment of the nodule and differentiating into an N_2_ fixing bacteroid [51,52], allowing them to maintain competitiveness during their free-living state in the soil. To mimic this oxygen-dependent regulation, we fused the *uAT-Ac2* allele to native or synthetic RBSs and placed these under control of the NifA-inducible P*nifH* promoter on mini-Tn*7* delivery plasmids, then integrated these into the chromosome of *Ac*LPΔ*glnE*, creating strains *Ac*PU-RStd, *Ac*PU-R1, *Ac*PU-R22, *Ac*PU-R31, *Ac*PU-Rnat and *Ac*PU-R28 (Fig 5A). When grown under aerobic (21% O_2_) conditions in the presence of 10 mM NH_4_Cl, growth of *Ac*LPΔ*glnE* expressing the *uAT*-*Ac2* allele from the non-induced P*nodA* promoter was almost entirely abolished compared to where glutamine was provided as a source of N (Figs 5B and S6). In contrast, the growth characteristics of strains expressing *uAT-Ac2* from the P*nifH* promoter were reminiscent of the wild-type *Ac*, except for strains where *uAT-Ac2* was fused to the strongest RBS’ RStd or R1, which increased mean generation times (MGT) but did not affect the total biomass at stationary phase (Figs 5B and S6). We next assessed GS regulation by γ-glutamyltransferase assays and confirmed that under aerobic conditions in the presence of 10 mM NH_4_Cl, the percentage of active deadenylylated GS activity in strains *Ac*PU-R1, *Ac*PU-R22, and *Ac*PU-R3 closely resembled that of the wild-type, suggesting that NH_3_ assimilation was functional. When grown under microaerobic conditions (3% O_2_) in the presence or absence of 10 mM NH_4_Cl, GS in wild-type *Ac*LP was activated by deadenylylation, whereas GS in all *Ac*LPΔ*glnE* strains expressing uAT-*Ac*2 from the P*nifH* promoter became more heavily inactivated by adenylylation under the same conditions (Fig 5C), with the percentage unadenylated GS activity correlating negatively with the strength of RBS fused to *uAT-Ac2*. We finally performed NH_3_ excretion assays on the engineered strains and found that each excreted NH_3_ into the growth media after 24-h, except for where *uAT-Ac2* was fused to the weakest RBS [R28] (Fig 5D). Overall, the data suggested that by expressing *uATs* from the P*nifH* promoter, GS shutdown could be controlled in response to atmospheric oxygen tension in a similar manner to the activation of N_2_-fixation.

### Rhizopine-dependent control of nitrogen fixation, GS adenylylation and ammonia excretion

**Fig 6:**
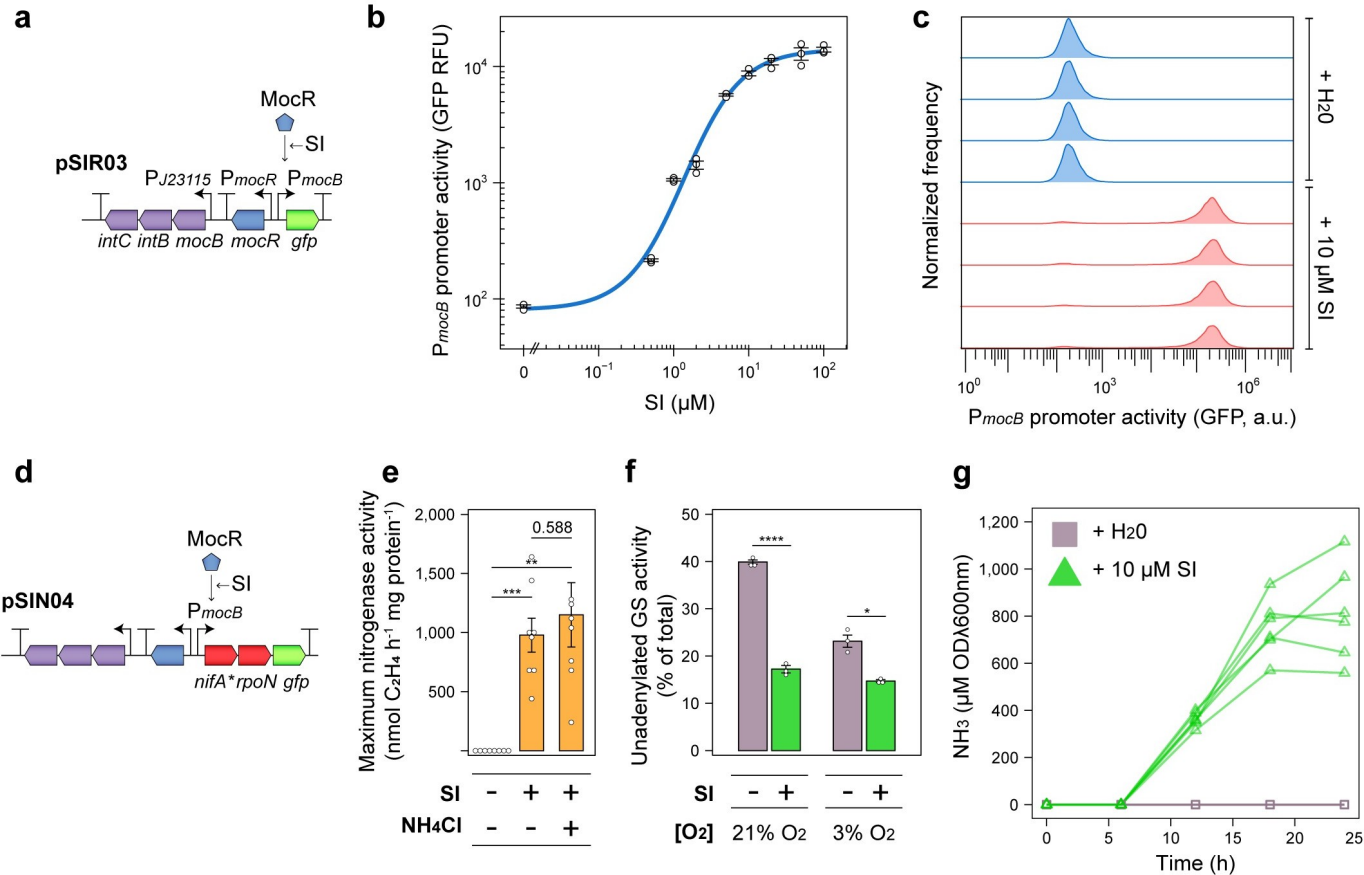
Rhizopine control of nitrogen fixation and ammonia excretion in *Ac*LP. **(a)** Genetic schematic (not to scale) of the low-copy (RK2 replicon) rhizopine receiver plasmid pSIR03. **(b)** Dose response of GFP induction in *Ac*LP (*n =* 3) harbouring pSIR03 with the rhizopine *scyllo*-inosamine (SI) supplemented *in vitro*. Relative fluorescence units (RFU) are defined here as GFP fluorescence/OD600λnm. **(c)** Flow-cytometry analysis of GFP fluorescence in *Ac*LP (*n =* 4) harbouring pSIR03 incubated for 24-h in the absence or presence of 10 μM rhizopine. See S1 Table for full statistics. **(d)** Genetic schematic (not to scale) of the low-copy (RK2 replicon) rhizopine *nifA*_*L94Q/D95Q*_*-rpoN* controller plasmid pSIN04. **(e)** Maximum nitrogenase activity of *Ac*PU-R22 *ΔnifA* carrying pSIN04 measured between 5-h– 21-h by acetylene reduction in *n =* 6 cultures grown under microaerobic conditions (3% O_2_ in the headspace). **(f)** Activity of the unadenylylated (active) form of GS in *n* = 3 cultures of *Ac*PU-R22 *ΔnifA* carrying pSIN04 incubated for 24-h as determined by γ-glutamyl transferase assays in the presence or absence of 60 mM MgCl_2_. **(g)** Spectrophotometric determination of NH_3_ in media of *n =* 6 cultures of *Ac*PU-R22*ΔnifA* carrying pSIN04 grown in N_2_-fixing conditions (N-free UMS media with 3% O_2_ in the headspace) in the presence of absence of 10 μM SI. Error bars represent one SEM. Independent two-tailed students t-tests with the Bonferroni-holm adjustment were used to compare means. Exact P-values are provided where P > 0.5. **P < 0.01, ***P < 0.001, ****P < 0.0001.

While NifA-dependent expression of nitrogenase and *uAT-Ac2* in *Ac*Δ*glnE* drives N_2_ fixation and GS inactivation leading to NH_3_ excretion, the lack of plant host-specific signalling to drive these processes could permit bacteria to supply NH_3_ to target crops and non-target weed species alike. We previously used synthetic rhizopine signalling to establish control of a mutant *nifA* allele (encoding NifA_L94Q/D95Q_) and *rpoN* in *Ac*LPΔ*nifA* carrying plasmid pSIN02, which drove partially NH_3_-resistant activation of nitrogenase activity specifically by bacteria occupying the roots of transgenic *RhiP* barley [47]. We performed NH_3_ excretion assays on *Ac*LPΔ*nifA* carrying pSIN02 and found that this strain did not secrete NH_3_ into the growth media (S7 Fig). Thus, we opted to establish rhizopine control of the *nifA*_*L94Q/D95Q*_*-rpoN* operon in our strain *Ac*PU-R22 where *uAT-Ac2* expression placed under control by NifA. We first tested in *Ac*LP, induction of a new rhizopine receiver plasmid pSIR03 which was derived from the high-copy rhizopine receiver pSIR03 but carried an RK2 replicon for more stable low-copy maintenance. Using *GFP* induction assays, we demonstrated that pSIR03 (Fig 6A) has a dynamic range of 162-fold in response to the rhizopine *scyllo-*inosamine (SI) and was induced in 93.08 ± [SEM] 0.32% of cells in populations when 10 μM SI was supplemented *in vitro* (Fig 6B and 6C and S1 Table). We deleted the native *nifA* gene from strain *Ac*PU-R22 and introduced a rhizopine *nifA*_*L94Q/D95Q*_*-rpoN* controller plasmid pSIN04 which was derived from pSIN03 (Fig 6D). Expression of *nifA*_*L94Q/D95Q*_*-rpoN* under microaerobic conditions by addition of 10 μM SI into the media resulted in tightly controlled activation of nitrogenase that was unimpeded by addition of 10 mM of NH_3_ (Fig 6E). Moreover, GS was strongly adenylylated by addition of 10 SI to the media in both aerobic and microaerobic conditions (Fig 6F). Because NifA in many diazotrophs is inactivated when cells are grown at 21% O_2_ [53], we subsequently tested O_2_ tolerance of our NifA_L94/D95Q_ mutant protein by inducing expression of *nifA*_*L94Q/D95Q*_*-rpoN* in *Ac*LPΔ*nifA* carrying pSIN03 with rhizopine and monitoring activation of the P*nifH*::*GFP* promoter fusion (S8A Fig). Interestingly, the NifA_L94/D95Q_ protein activated P*nifH*::*GFP* 13-fold ± [SEM] 1.5 and 98-fold ± [SEM] 2.8 under aerobic and microaerobic conditions, respectively (S8B Fig), suggesting that the protein is tolerant to oxygen.

We next assessed growth of our engineered strain *Ac*PU-R22Δ*nifA* carrying pSIN04 where NH_4_Cl was provided as a sole source of N (S9 Fig). As expected, growth was strongly inhibited in the presence of 10 μM SI, indicating that the strain was unable to assimilate NH_3_ in this state. When grown in the absence of nitrogen under N_2_-fixing conditions, the strain excreted 812.58 ± [SEM] 5.59 uM ODλ600nm^-1^ NH_3_ into the media after 24-h incubation at an optimal rate of 65.13 ± 7.35 uM OD600nm^-1^ h^-1^ (Fig 6G). These experiments confirmed that we had established tight rhizopine control of N_2_-fixation, GS adenylylation and NH_3_ excretion in our engineered *Ac*LP strain.

## Discussion

In this study, we employed two strategies to interfere with GS and stimulate NH_3_ excretion in *Ac*LP. For our first strategy, we attempted to recapitulate previous experiments where insertional inactivation of the *P*_*II*_ genes *glnB* and *glnK* stimulated shutdown of GS by adenylylation and alleviated negative feedback inhibition of nitrogenase by the product NH_3_, preventing NH_3_ assimilation and favouring excretion into the growth media [29]. Although we could delete either of the *glnB* or *glnK* genes from *Ac*LP, we were unable to delete both genes in the same strain unless a second copy of *glnB* was first introduced into the chromosome, suggesting at least one of the P_II_ coding sequences was essential for growth. Considering that a P*aph*::KIXX kanamycin resistance cassette was previously inserted to the 3’-end of the *Ac glnB* coding sequence [26] leaving most of the 5’-end reading frame intact, it seems possible that the GlnB protein may have retained some essential function unrelated to AT and GS activity. In contrast, previous insertion of the omega interposon into *Ac glnK* replaced a segment of the internal coding sequence and was therefore more likely to have abolished the function of the protein [54]. Interestingly, similar *glnB* and *glnK* antibiotic cassette insertions have been made in the phototrophic diazotroph *Rhodobacter capsulatus*, resulting in NH_3_-insensitive NifA and nitrogenase expression and activity [55]. However, attempts to delete both genes were also unsuccessful in this bacterium [56]. Regardless of why deleting *glnB* and *glnK* is lethal, reproducing exact copies of the original *glnB* and *glnK* mutants [29] would likely be required to establish control of NH_3_ excretion in *Ac*LP, as we have shown here that deletion of *glnK* paired with strong repression of *glnB* had minimal effect on nitrogenase or GS regulation and permitted only low-level NH_3_ excretion.

As was previously demonstrated in *A*. *brasilense* [30], expression of *E*. *coli* or *Ac*-derived uATs in our *Ac*LPΔ*glnE* mutant resulted in strong GS shutdown and high rates of NH_3_ excretion when grown in N_2_-fixing conditions. We also found that while nitrogenase expression and activity is repressed in microaerobic NH_3_ or glutamine-fed cultures of *Ac*LP, shutdown of glutamine biosynthesis by uAT expression resulted in nitrogenase expression that was unimpeded by NH_3_ but still repressed by glutamine, suggesting that NH_3_ must first be converted to glutamine or potentially other amino acids such as asparagine [57] to facilitate repression. This same effect was previously reported in phototrophic *Anabaena* spp [58,59] and *Rhodobacter sphaeroides* [60] where GS activity was shutdown using the chemical inhibitor L-Methionine sulfoximine, and in *Klebisella pneumoniae* mutants unable to grow on NH_3_ as a sole source of N [61]. Moreover, In *R*. *capsulatus*, where N_2_-fixation is repressed in response to added NH_3_ at three levels; a) NtrC-dependent transcription of *nifA*; b) NifA-dependent transcription of nitrogenase; and c) DraT-DraG-dependent ADP ribosylation of nitrogenase [62,63]; all three levels of regulation were non-responsive to NH_3_ following shutdown of GS by insertional inactivation of both P_II_ genes [55]. Thus, it seems plausible that shutdown of glutamine biosynthesis from NH_3_ and glutamate abolishes NH_3_-dependent regulation of N_2_-fixation in genetically diverse bacteria. Targeted GS shutdown therefore affects NH_3_ excretion on two fronts, allowing sustained nitrogenase expression and activity in the presence of fixed N_2_ and preventing assimilation of NH_3_, favouring excretion into the environment.

Without establishing control of GS shutdown, engineered NH_3_ excreting diazotrophs are typically auxotrophic for glutamine, which would render them non-competitive in the environment [14,17]. Here, we placed expression of the *uAT-Ac2* allele under control of the NifA-inducible nitrogenase promoter P*nifH* which, when tuned correctly, triggered GS shutdown and NH_3_ excretion specifically under N_2_-fixing conditions. In the field, this could allow bacteria to retain competitiveness prior to forming oxygen-deplete biofilms on the surface of roots [64], however lack of host-specific control could permit provision of NH_3_ to non-target plant species. Thus, we further modified the engineered strain *Ac*PU-R22 by deleting *nifA* and bringing the mutant *nifA*_*L94Q/D95Q*_ and *rpoN* alleles under rhizopine-inducible control, permitting *in vitro* rhizopine-dependent activation of nitrogenase activity, GS shutdown and NH_3_ excretion. In future, we aim to further demonstrate activation of these processes by the bacteria colonising the roots or rhizosphere of transgenic rhizopine producing (*RhiP*) barley [48], though we acknowledge that this will first require optimisation of rhizopine perception by *Ac*LP carrying a rhizopine biosensor plasmid. At present, current rhizopine biosensors only permit perception of rhizopine by 10–25% of cells colonising *RhiP* barley roots, and in congruency, activation of *in situ* nitrogenase activity in these populations amounts to approximately 15% of that observed in wild-type *Ac*LP cells colonising wild-type barley [47]. In addition to improving rhizopine perception, developing strategies to stabilise function of the engineered genes *in situ* will be crucial to generate a practical interaction between the bacteria and plants.

Although we have demonstrated controlled activation of N_2_ fixation and NH_3_ excretion in response to rhizopine, it remains likely that increased energy demand in this state might be detrimental to viability and competitiveness on the root surface [15]. Rhizobia overcome this problem by engaging in stringent signalling with the legume that permits partner-specific infection of nodules [65]. Inside the nodule, the bacteria are provided with low-oxygen conditions conducive to nitrogenase stability, they can escape the fierce competition of the rhizosphere, and are fed carbon in the form of dicarboxylates [51,52]. Engineering a nodule-like niche with stringent entry requirements into cereals will likely be important to maximise the effectiveness of inoculation with engineered NH_3_ excreting inoculants. The strains developed here could be adapted for entry of such an environment and therefore, this work represents significant advancement towards the development of both associative and more intimate “synthetic N_2_-fixing symbiosis” with cereals.

## Materials and methods

### Bacterial strains and plasmids

Bacteria used in this study (S1 File) were cultured in TY [66] or UMS [67,68] media supplemented with 300 μM nicotinic acid and 20 mM succinate as previously described [47]. Plasmids (S2 Table) were constructed using HiFi assembly (New England Biolabs) or BEVA modular golden-gate assembly [69,70] as outlined in the S1 File and were mobilised into *Azorhizobium* by diparental mating with *E*. *coli* ST18 [71]. For mini-Tn*7* integration into the chromosome, tri-parental matings were used to additionally mobilise the transposase helper plasmid pTNS3, which carries an R6K origin of replication that is not maintained in *Azorhizobium* [72].

Gene deletion and replacement mutant strains were constructed by mobilising the relevant suicide plasmid, derived from pK19mobSacB (S2 Table and S1 File), into the target strain and selecting for single-crossover integration into the chromosomal region of interest by plating cells on selective UMS or TY agar media supplemented with 100 μg mL^-1^ kanamycin. Single-crossover mutants were subsequently grown in non-selective media until stationary phase and plated in serial dilutions onto UMS or TY agar supplemented with 10% (v/v) sucrose to select for double crossover deletion or replacement of the target gene. For the *ΔglnK*::Ω*Sp* replacement plasmid pOPS1564 only, 100 μg mL^-1^ spectinomycin and 1 mM IPTG was added to the media unless otherwise stated. Single colonies were patched onto the same media used for double-crossover selection plus and minus 100 μg mL^-1^ kanamycin and kanamycin sensitive colonies were screened by PCR and sanger sequencing for deletion or replacement of the target gene.

All *Ac*LP *ΔglnB ΔglnK*::Ω*Sp* mutant strains were constructed by first deleting *glnB* from *Ac*LP using plasmid pOPS1691, then subsequently integrating the *ΔglnK*::Ω*Sp* replacement plasmid pOPS1564 into the target chromosomal region by single-crossover. Because replacement of *ΔglnK*::Ω*Sp* was not possible on three separate occasions, mini-Tn*7* delivery plasmids carrying an IPTG-derepressible copy of *glnB* (S1 File) were integrated into the engineered *attB* site prior to selecting for selecting for double-crossover replacement of *glnK* with the Ω*Sp* interposon as described above.

### Growth curves

Growth curves were performed in triplicate by streaking single colonies onto 10 mL TY agar slopes and incubating for 3-days prior to three washes in PBS and inoculation at OD600λnm 0.01 into 500 μL UMS media in 24-well plates. The OD600λnm was monitored at 20 min intervals in an Omega FLUOstar plate reader set to shake cultures at 700 rpm at 37°C until stationary phase. Growth statistics were calculated using the R package GrowthCurver [73].

### GS transferase assays

Six-millilitre UMS cultures were initially grown in 30 mL glass universal vials sealed with silicone rubber septa as described for RT-qPCR experiments. After 3-h or 24-h incubation, 1 mL of culture was sampled for protein determination using a Millipore BCA protein assay kit. Five hundred microlitres of CTAB (1 mg mL^-1^) was added to the remaining cultures which were incubated at room temperature for a further 3 mins prior to harvesting by centrifugation at 4°C. Cells were washed once with 5 mL 1% (w/v) KCL and finally resuspended in 500 μL of the same buffer and stored on ice. GS transferase assays were performed on 50 μL aliquots the permeabilized cells as previously described [16]. The assays were performed in 500 μL total volumes with 30 min incubation in the presence or absence of 60 μM added Mg_2_Cl to determine the total GS transferase activity and the activity of the “active” unadenylylated enzyme, respectively [49]. The GS transferase buffer was adjusted to pH 7.0, as this was previously estimated as the iso-activity point for *Ac* [74]. Following addition of the FeCl_3_ stop reagent, reaction tubes were centrifuged for 5 min at 13,000 g and 200 μL was transferred to clear, flat bottomed 96-well plates for spectrophotometric quantification of the product L-Glutamyl-γ-Hydroxamate (LGH) at 562λnm in a Promega GloMax multi-detection system.

### Acetylene reduction assays

Cultures for ARAs were prepared and analysed as previously described [47, 75] and 1 mL samples of the headspace atmosphere were analysed using a PerkinElmer Clarus 480 gas chromatograph equipped with a HayeSep N (80–100 MESH) 584 column at 3-h, 5-h, 21-h, 23-h and 25-h incubation, unless otherwise stated.

### Ammonia excretion assays

Three-millilitre UMS cultures were initially grown in 30 mL glass universal vials sealed with silicone rubber septa as described for RT-qPCR experiments. OD600λnm was recorded and NH_3_ was quantified in spent supernatants using the spectrophotometric indophenol assay as previously described [16]. A calibration curve was performed for each experiment using freshly made dilutions of NH_4_Cl in UMS ranging from 5 μM– 1 mM. Absorbance of indophenol blue was quantified in a Genesys 150 UV visible spectrophotometer (Thermo Scientific) at 652λnm after 4-h incubation at room temperature.

### RT-qPCR

For RT-qPCR experiments, *n =* 5 single colonies were streaked onto 10 mL UMS agar slopes supplemented with 20 mM succinate, 10 mM NH_4_Cl and 300 μM nicotinate and grown for 2-days at 37°C. Cells were washed three times from the slopes with PBS, resuspended in UMS supplemented with the relevant carbon and N sources at OD600λnm 0.3 in 30 mL glass universal vials and transferred with the lid off into a sealed atmosphere cabinet adjusted to 3% O_2_ by flushing with N_2_ gas. After 30 min, cultures were sealed with silicone rubber septa and incubated at 37°C with rigorous shaking for 3-h. Cells were next harvested by centrifugation at 4°C, lysed using a FastPrep-24 5G instrument and cellular debris was removed by a second round of centrifugation. RNA was extracted from the resulting lysate using a Qiagen RNAeasy extraction kit and tested for quality and purity using an Agilent Experion Bioanalyzer with RNA Stdsens chips. gDNA was depleted from RNA by treatment with Invitrogen Turbo DNAse as per the manufacturer’s recommendations and 5 μg was used to generate cDNA using an Invitrogen SuperScript IV reverse transcriptase kit as per the manufacturer’s recommendations. The final cDNA template was diluted 1:20 with water and 1 μL was added to each 20 μL RT-qPCR reaction prepared in 96-well plates with Applied Biosystems PowerUp SYBR green master mix. Reactions were run using an Applied Biosystems ViiA 7 Real-Time PCR system. RT-qPCR primers were initially tested for amplification efficiency and target specificity by generating a standard curve of amplification with 5-fold dilutions of *Ac*LP gDNA. The housekeeping gene primer targeted *recA* and was validated previously [76], whereas the *glnA* primers designed here had the following sequence *glnA F* 5’- CCGCTGACCAACTCCTACA *glnA R* 5’- CCATGAACAGGGCCGAGAA.

### GFP reporter assays and flow-cytometry

GFP reporter assays and flow-cytometry experiments were performed on 24-h incubated cultures as previously described [47]. Inducers were added directly to the growth media at the time of inoculation where relevant.

## Supporting information

S1 Fig Characterisation of synthetic ribosome binding sites in *Ac*LP. Each RBS was fused to GFP under expression by the strong synthetic promoter J23104 on plasmid pOGG024 and GFP was measured after 24-h incubation in UMS media (*n* = 3). Relative luminescence units are defined here as GFP fluorescence/OD600λnm. The RBS nucleotide sequences are provided in S1 File.
(TIF)

S2 Fig Expression and total activity of GS is elevated in *Ac*RGl. **(a)** Total specific activity of both adenylated (inactive) and unadenylated (active) forms of GS was measured in whole cells grown for 24-h as determined by γ-glutamyl transferase assays (*n =* 5). **(b)** *glnA* expression was quantified relative to the housekeeping gene *recA* by RT-qPCR in cells growth for 3-h. All cultures for assays were grown in N_2_-fixing conditions (N-free UMS media with 3% O_2_ in the headspace). Error bars represent one SEM. Independent two-tailed students t-tests were used to compare means. ***P < 0.001.
(TIF)

S3 Fig Induction of the *Sinorhizobium meliloti* 1021 naringenin-inducible P*nodA* promoter in *Ac*LP (a) Genetic schematic (not to scale) of the low-copy (RK2 replicon) naringenin-inducible *GFP* reporter plasmid pOPS1536. (b) GFP induction in *Ac*LP (*n* = 3) harbouring pOPS1536 in response to naringenin supplemented *in vitro*. Relative luminescence units are defined here as GFP fluorescence/OD600λnm.
(TIF)

S4 Fig Total activity of GS in *ΔglnE* mutants expressing uATs from the non-induced P*nodA* promoter (a) Total specific activity of both adenylated (inactive) and unadenylated (active) forms of GS was measured in whole cells grown in N_2_-fixing conditions (N-free UMS media with 3% O_2_ in the headspace) for 3-h as determined by γ-glutamyl transferase assays (*n* = 5 for wild-type *Ac*LP or *n* = 3 for other strains). Error bars represent one SEM. Independent two-tailed students t-tests were used to compare means against the wild-type (WT) *Ac*LP as a reference. Not significant (ns) indicates P > 0.05, *P < 0.05.
(TIF)

S5 Fig Ammonia excretion is suboptimal in *ΔglnE* mutants expressing uATs from the P*nodA* promoter induced with naringenin. Spectrophotometric determination of NH_3_ in media of cultures induced with 5 μM naringenin grown for 24-h in N_2_-fixing conditions (N-free UMS media with 3% O_2_ in the headspace). Error bars represent one SEM. *n =* 3 for wild-type *Ac*LP *ΔglnE* or *n* = 6 for other strains.
(TIF)

S6 Fig Growth statistics for control strains and *ΔglnE* mutants expressing uATs. Mean generation times and the max OD600λnm (i.e. the carrying capacity, k) were calculated from standard curves of cultures grown in UMS media at 21% O_2_. Strains highlighted in white are wild-type (WT) *Ac*LP and *Ac*LPΔ*glnE* controls, strains highlighted in pink are *Ac*LPΔ*glnE* carrying P*nodA* [RBS] uAT-DT16 modules on parent plasmid pOGG093 and strains highlighted in blue are *Ac*LPΔ*glnE* carrying mini-Tn*7* integrated P*nifH* [RBS] uAT-*Ac2*-DT16 modules.
(TIF)

S7 Fig Rhizopine control of nitrogen fixation alone does not permit ammonia excretion. Spectrophotometric determination of NH_3_ in media of *n =* 3 cultures grown for 24-h in N_2_-fixing conditions. Error bars represent one SEM. Strain *Azospirillum brasilense* HM053 was used here as a positive control.
(TIF)

S8 Fig NifA_L94Q/D95Q_ activity is tolerant to ambient environmental oxygen tensions. **(a)** Genetic schematic (not to scale) of the rhizopine *nifA*_*L94Q/D95Q*_-*rpoN* controller plasmid with P*nifH*::*GFP* reporter fusion pSIN03. **(b)** P*nifH* promoter activity was measured in *n =* 4 cultures grown for 24-h under the conditions indicated. Relative fluorescence units (RFU) are defined here as GFP fluorescence/OD600λnm. Error bars represent one SEM. Independent two-tailed students t-tests with Bonferroni-holm adjustment were used to compare means. P > 0.05. **P < 0.01, ***P < 0.001.
(TIF)

S9 Fig Growth statistics for *Ac*PU-R22Δ*nifA* carrying pSIN04. Growth of treatment and control strains was assessed in UMS media supplemented with 10 mM NH_4_Cl as a sole source of N and in the presence of absence of 10 μM of the rhizopine *scyllo-*inosamine (SI) **(a)** Growth curves are representative of *n = 3* replicates per treatment condition. **(b)** Mean generation times (MGTs) were calculated from the growth curves using Growthcurver [73]. Error bars represent one SEM. Independent two-tailed students t-tests were used to compare means. Exact P values are provided where P > 0.05. *P < 0.05.
(TIF)

S1 Table Flow-cytometry statistics for rhizopine-inducible GFP expression in *Ac*LP carrying pOPS1052. (DOCX)

S2 Table Plasmids used in this study. (DOCX)

S1 File Bacterial strains, golden-gate pieces, plasmid construction and oligonucleotides. (XLSX)
