# Supplementary figures and images for: Control of nitrogen fixation and ammonia excretion in Azorhizobium caulinodans
Source: PLoS Genet. 2022 Jun 21;18(6):e1010276. doi: 10.1371/journal.pgen.1010276 (PMC9249168; doi:10.1371/journal.pgen.1010276)

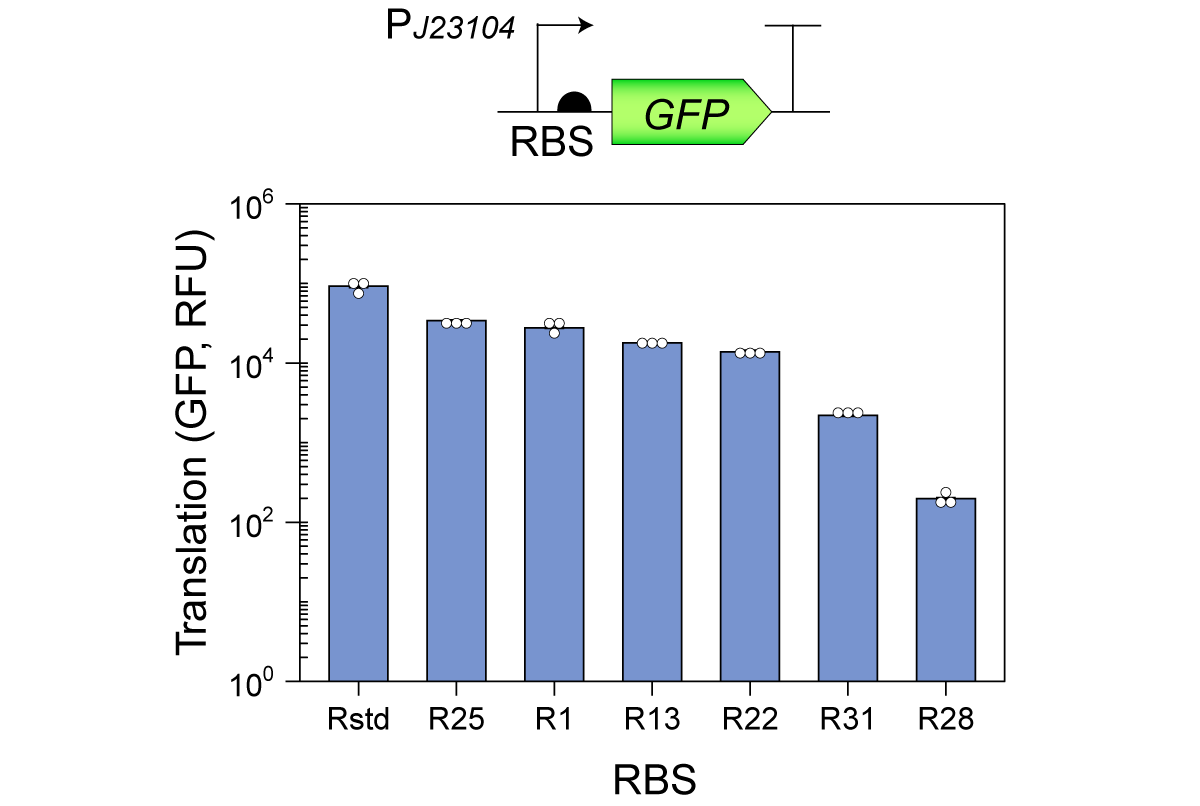

Supplement: S1 Fig — Each RBS was fused to GFP under expression by the strong synthetic promoter J23104 on plasmid pOGG024 and GFP was measured after 24-h incubation in UMS media (n = 3). Relative luminescence units are defined here as GFP fluorescence/OD600λnm. The RBS nucleotide sequences are provided in S1 File. (TIF) [file pgen.1010276.s001.tif]

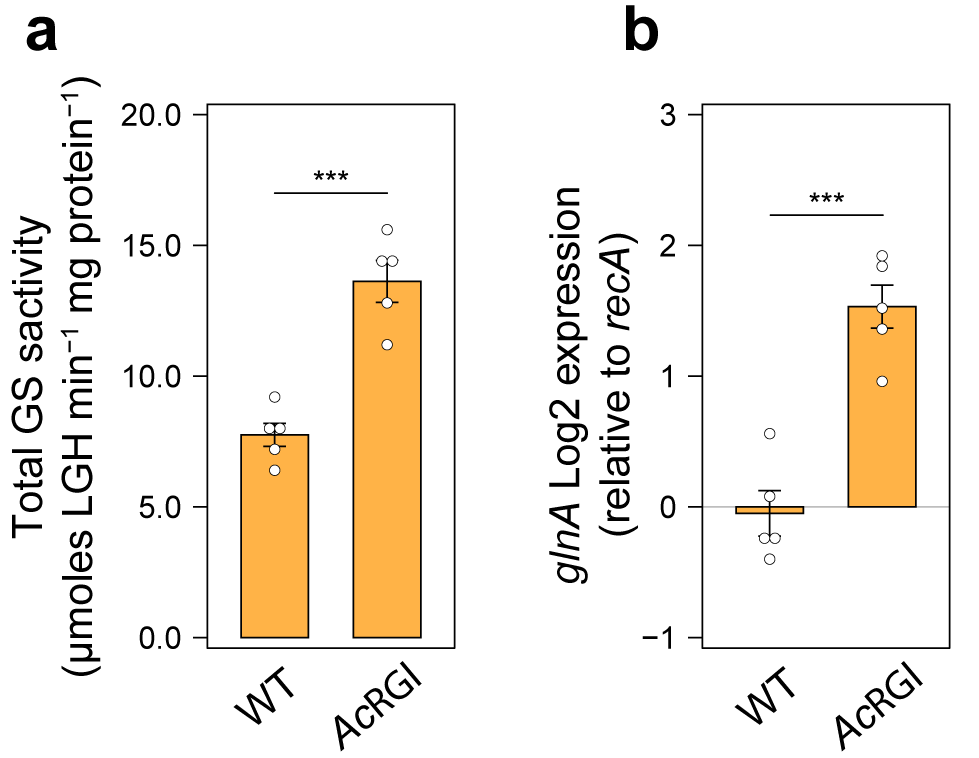

Supplement: S2 Fig — (a) Total specific activity of both adenylated (inactive) and unadenylated (active) forms of GS was measured in whole cells grown for 24-h as determined by γ-glutamyl transferase assays (n = 5). (b) glnA expression was quantified relative to the housekeeping gene recA by RT-qPCR in cells growth for 3-h. All cultures for assays were grown in N2-fixing conditions (N-free UMS media with 3% O2 in the headspace). Error bars represent one SEM. Independent two-tailed students t-tests were used to compare means. ***P < 0.001. (TIF) [file pgen.1010276.s002.tif]

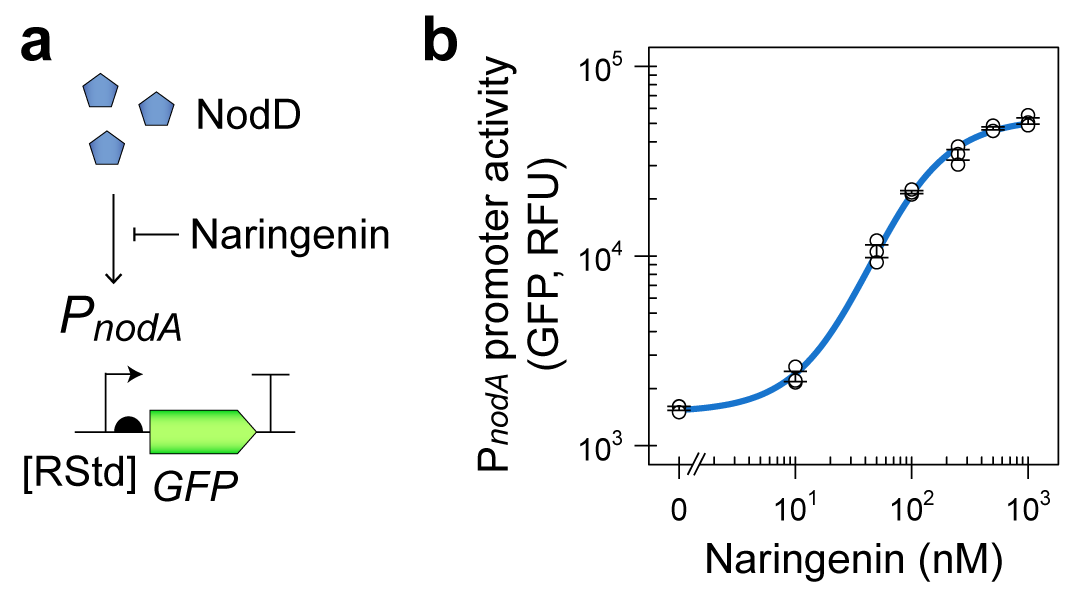

Supplement: S3 Fig — Induction of the Sinorhizobium meliloti 1021 naringenin-inducible PnodA promoter in AcLP (a) Genetic schematic (not to scale) of the low-copy (RK2 replicon) naringenin-inducible GFP reporter plasmid pOPS1536. (b) GFP induction in AcLP (n = 3) harbouring pOPS1536 in response to naringenin supplemented in vitro. Relative luminescence units are defined here as GFP fluorescence/OD600λnm. (TIF) [file pgen.1010276.s003.tif]

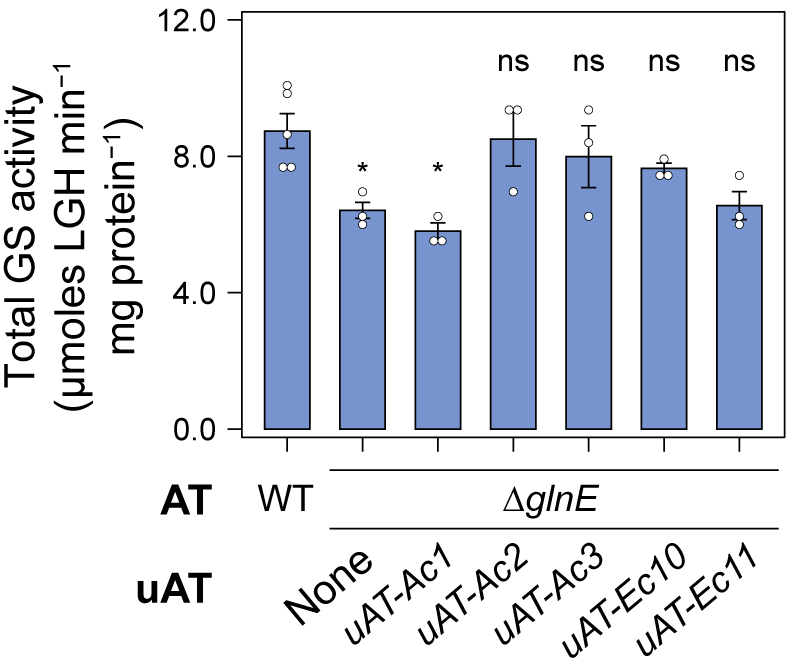

Supplement: S4 Fig — Total activity of GS in ΔglnE mutants expressing uATs from the non-induced PnodA promoter (a) Total specific activity of both adenylated (inactive) and unadenylated (active) forms of GS was measured in whole cells grown in N2-fixing conditions (N-free UMS media with 3% O2 in the headspace) for 3-h as determined by γ-glutamyl transferase assays (n = 5 for wild-type AcLP or n = 3 for other strains). Error bars represent one SEM. Independent two-tailed students t-tests were used to compare means against the wild-type (WT) AcLP as a reference. Not significant (ns) indicates P > 0.05, *P < 0.05. (TIF) [file pgen.1010276.s004.tif]

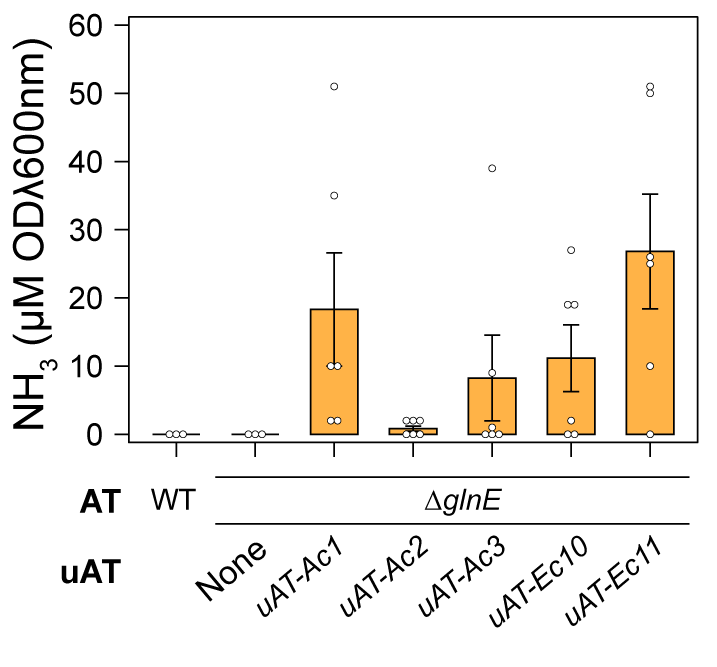

Supplement: S5 Fig — Spectrophotometric determination of NH3 in media of cultures induced with 5 μM naringenin grown for 24-h in N2-fixing conditions (N-free UMS media with 3% O2 in the headspace). Error bars represent one SEM. n = 3 for wild-type AcLP ΔglnE or n = 6 for other strains. (TIF) [file pgen.1010276.s005.tif]

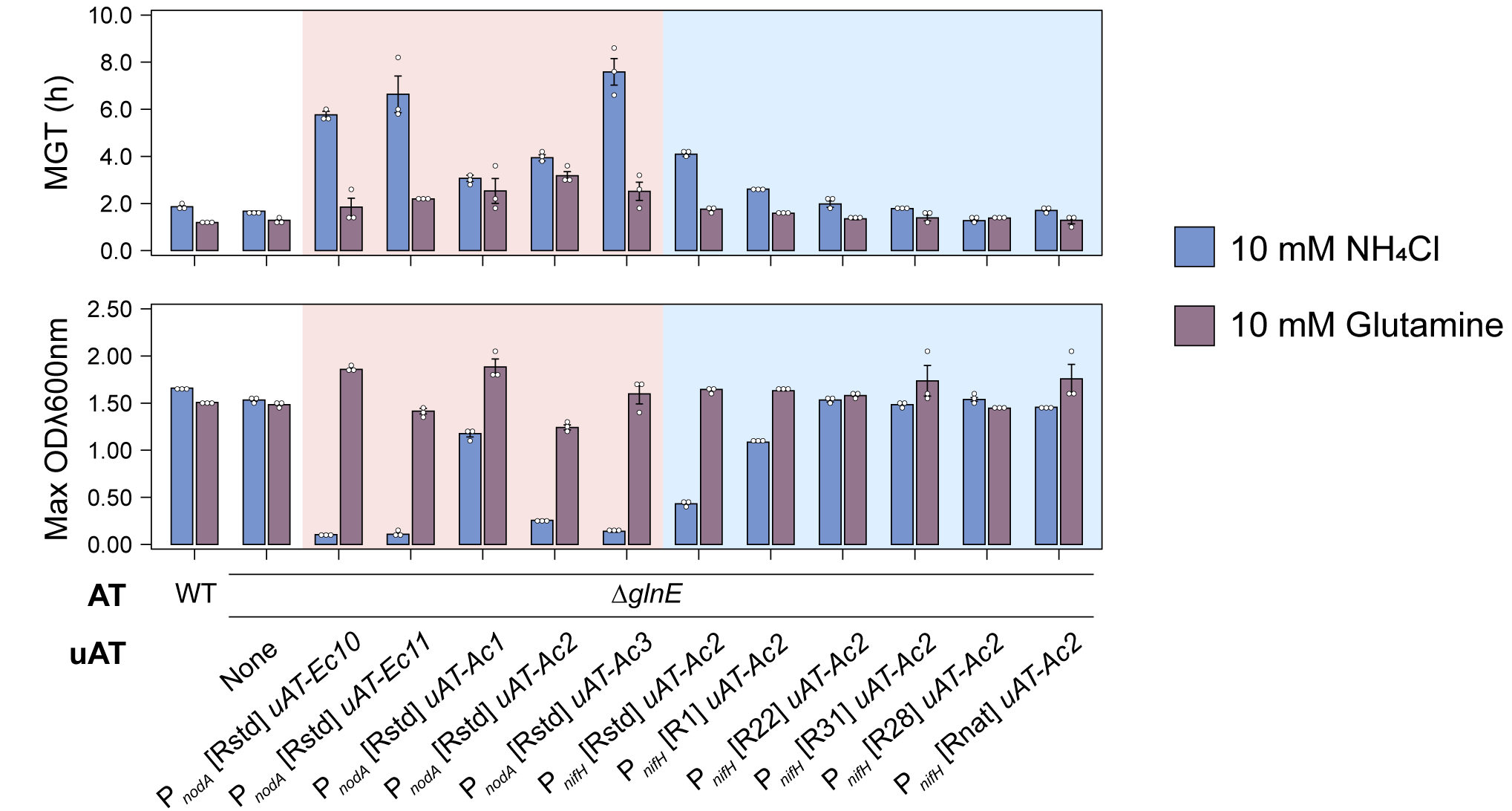

Supplement: S6 Fig — Mean generation times and the max OD600λnm (i.e. the carrying capacity, k) were calculated from standard curves of cultures grown in UMS media at 21% O2. Strains highlighted in white are wild-type (WT) AcLP and AcLPΔglnE controls, strains highlighted in pink are AcLPΔglnE carrying PnodA [RBS] uAT-DT16 modules on parent plasmid pOGG093 and strains highlighted in blue are AcLPΔglnE carrying mini-Tn7 integrated PnifH [RBS] uAT-Ac2-DT16 modules. (TIF) [file pgen.1010276.s006.tif]

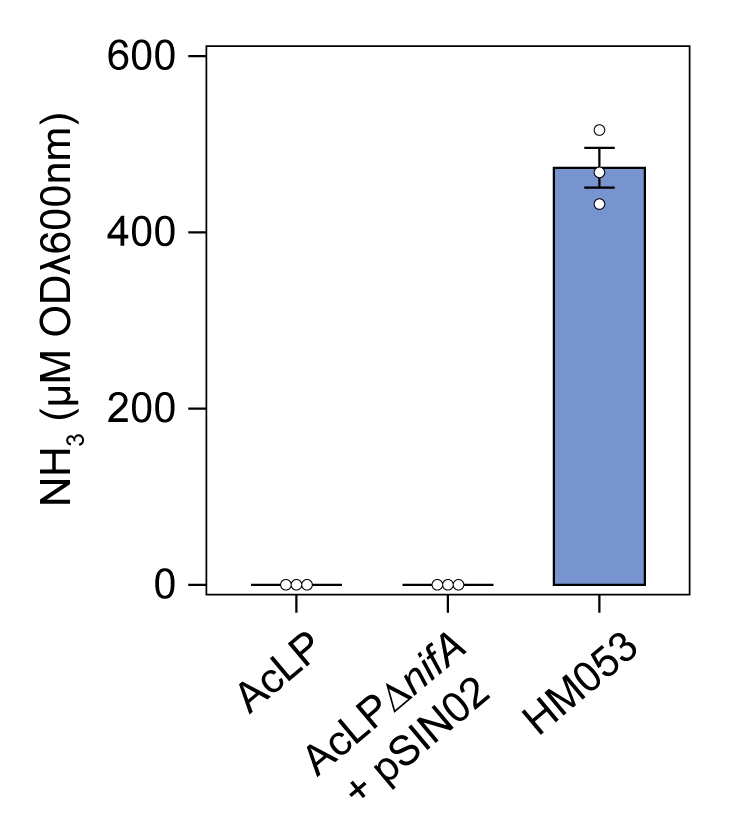

Supplement: S7 Fig — Spectrophotometric determination of NH3 in media of n = 3 cultures grown for 24-h in N2-fixing conditions. Error bars represent one SEM. Strain Azospirillum brasilense HM053 was used here as a positive control. (TIF) [file pgen.1010276.s007.tif]

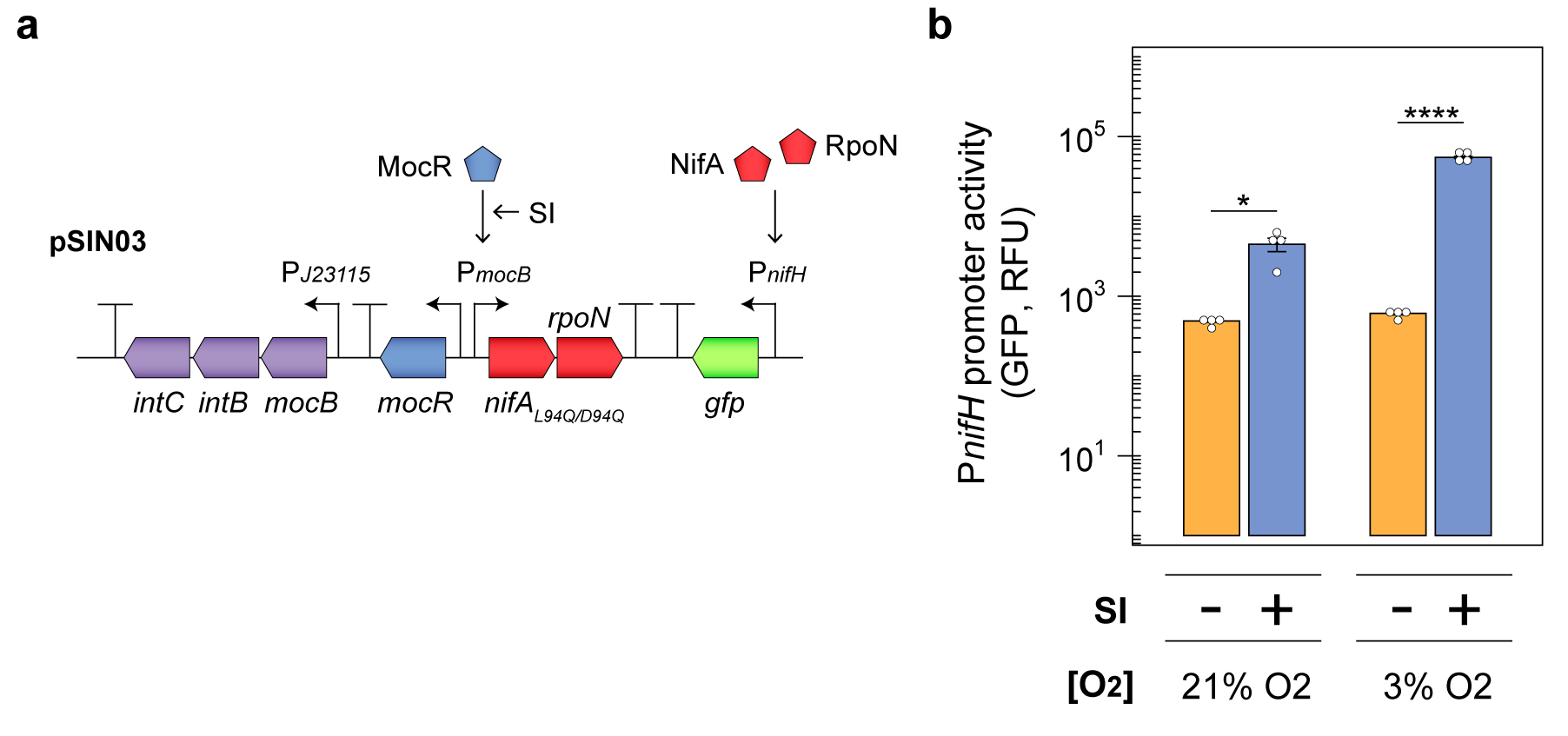

Supplement: S8 Fig — (a) Genetic schematic (not to scale) of the rhizopine nifAL94Q/D95Q-rpoN controller plasmid with PnifH::GFP reporter fusion pSIN03. (b) PnifH promoter activity was measured in n = 4 cultures grown for 24-h under the conditions indicated. Relative fluorescence units (RFU) are defined here as GFP fluorescence/OD600λnm. Error bars represent one SEM. Independent two-tailed students t-tests with Bonferroni-holm adjustment were used to compare means. P > 0.05. **P < 0.01, ***P < 0.001. (TIF) [file pgen.1010276.s008.tif]

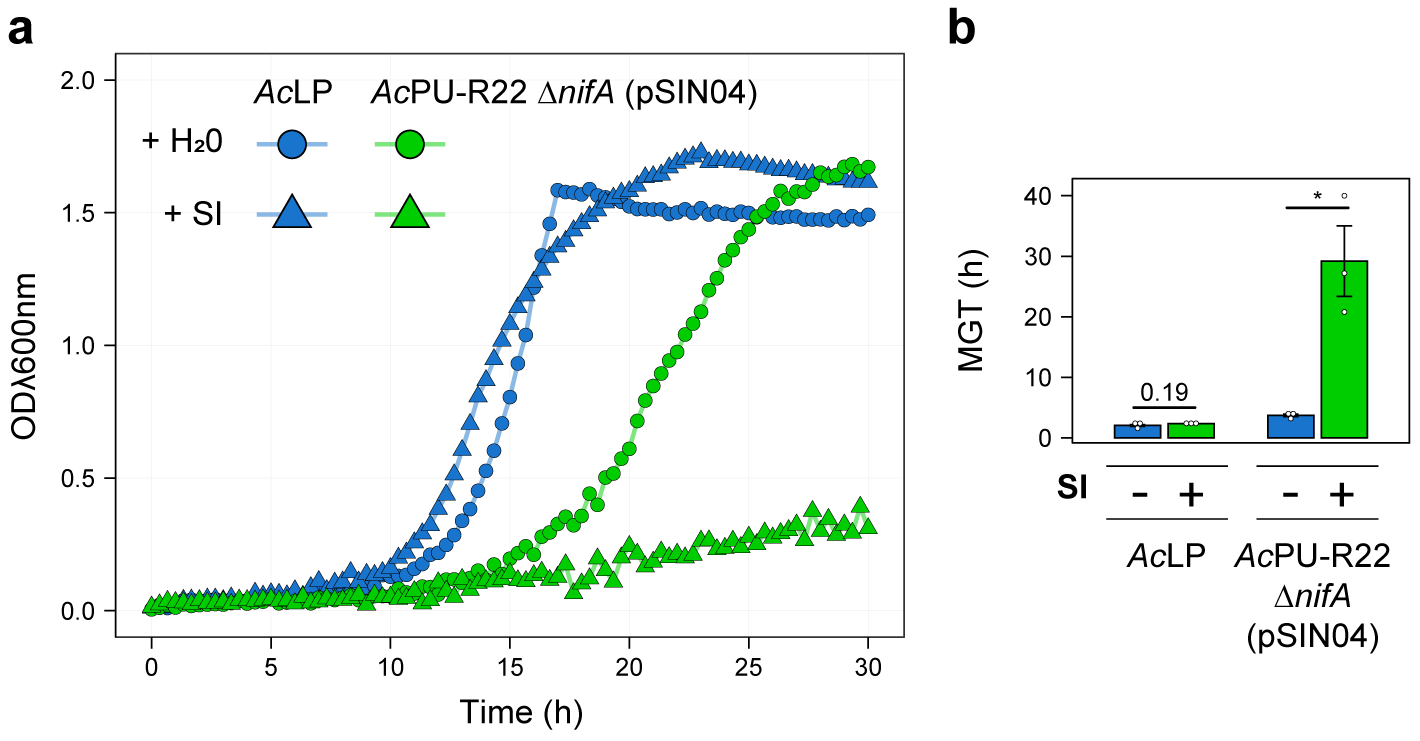

Supplement: S9 Fig — Growth of treatment and control strains was assessed in UMS media supplemented with 10 mM NH4Cl as a sole source of N and in the presence of absence of 10 μM of the rhizopine scyllo-inosamine (SI) (a) Growth curves are representative of n = 3 replicates per treatment condition. (b) Mean generation times (MGTs) were calculated from the growth curves using Growthcurver [73]. Error bars represent one SEM. Independent two-tailed students t-tests were used to compare means. Exact P values are provided where P > 0.05. *P < 0.05. (TIF) [file pgen.1010276.s009.tif]
